# Impact of Body Mass Index on In-Hospital Outcomes After Transcatheter Aortic Valve Replacement: A Retrospective Cohort Study from Saudi Arabia

**DOI:** 10.3390/life16010150

**Published:** 2026-01-16

**Authors:** Fawaz Khateb, Yosra A. Turkistani, Abdullah F. Rawas, Mustafa A. Sunbul, Abdullah Ghabashi, Ismail Alghamdi, Saleh M. Khouj

**Affiliations:** 1 Department of Medicine, College of Medicine, Umm Al-Qura University, Makkah 24382, Saudi Arabia; fawazrk@outlook.com (F.K.); afrws22@gmail.com (A.F.R.); mu96afa_72@hotmail.com (M.A.S.); 2 Department of Cardiology, King Abdullah Medical City, Makkah 24246, Saudi Arabia

**Keywords:** aortic stenosis, transcatheter aortic valve replacement, body mass index, outcomes, obesity paradox

## Abstract

Body mass index (BMI) has shown inconsistent associations with outcomes after transcatheter aortic valve replacement (TAVR), and evidence from the Middle Eastern population is limited. This study evaluated whether BMI independently predicts early complications, mortality, or infection following TAVR in a Saudi Arabian cohort. We conducted a retrospective analysis of 197 patients who underwent TAVR between 2015 and 2024, stratified by BMI < 25, 25–29.9, and ≥30 kg/m^2^. The primary endpoint was the in-hospital Valve Academic Research Consortium-3 (VARC-3) composite safety outcome, with secondary outcomes including individual complications, infection, length of stay, and 30-day mortality or readmission. Overall, patients had a mean age of 74.9 ± 8.8 years and 52.3% were female; in-hospital mortality was 2.0%, technical success 99%, and 30-day readmission 12.7%. BMI category was not independently associated with in-hospital complications or mortality, while advanced age ≥ 75 years (adjusted OR 2.52, *p* = 0.009), moderate Society of Thoracic Surgeons (STS) risk (adjusted OR 3.75, *p* = 0.008), and high STS risk (adjusted OR 2.26, *p* = 0.033) independently predicted complications. Overweight patients had higher in-hospital infection rates (14.1% vs. ~3%, *p* = 0.020). These findings suggest that physiologic vulnerability and operative risk, rather than BMI alone, should guide early TAVR risk assessment.

## 1. Introduction

Severe aortic stenosis (AS) is common in older adults and, once symptomatic, carries a poor prognosis without valve replacement. Transcatheter aortic valve replacement (TAVR) has transformed care across surgical risk categories, demonstrating non-inferiority or superiority to surgery in randomized trials and expanding TAVR into intermediate and low-risk populations [1,2,3]. Despite these advances, outcomes after TAVR vary with patient factors, warranting evaluation of modifiable and non-modifiable risks.

Body mass index (BMI) is one debated risk factor. Although obesity increases general cardiovascular risk, multiple TAVR series and meta-analyses describe an apparent “obesity paradox,” in which overweight and mildly obese patients have similar or better short- and mid-term outcomes than normal-weight peers [4,5,6,7,8]. Some analyses, however, suggest any protective effect of higher BMI may attenuate or reverse at extreme obesity, and they consistently report that underweight status portends worse prognosis (often viewed as a marker of frailty and malnutrition) [6,7,8]. Moreover, most prior TAVR studies derive from North American or European cohorts; the generalizability to regions with different BMI distributions, such as the Middle East, which has among the highest obesity prevalence in the world [9], remains uncertain.

Against this backdrop, we examined the association between BMI and post-TAVR outcomes in a contemporary Saudi Arabian cohort. Using standardized endpoints based on Valve Academic Research Consortium-3 (VARC-3) definitions [10,11], we hypothesized that BMI would not independently predict in-hospital major complications or mortality, and we additionally examined whether postoperative infection rates differ by BMI category. Through this study, we aim to clarify the clinical relevance of BMI in patient selection, peri-procedural management, and follow-up within an understudied population.

## 2. Materials and Methods

This retrospective observational cohort included all 197 consecutive patients who underwent TAVR at King Abdullah Medical City (KAMC), Makkah, between 1 January 2015 and 31 January 2024. Patients were included regardless of surgical risk category or access route; the vast majority underwent transfemoral TAVR. (Only one patient had an alternative access, subclavian, as noted below.) Patients without a recorded BMI were to be excluded, but all patients had BMI documented, so none were excluded for this reason. No additional exclusion criteria were applied.

We collected patient demographics, cardiovascular risk factors (e.g., diabetes, hypertension, dyslipidemia), prior cardiac history (including prior percutaneous coronary intervention (PCI) and coronary artery bypass grafting (CABG), medication history (antiplatelet/anticoagulant use), and baseline echocardiographic parameters [left ventricular ejection fraction (LVEF), annulus dimensions] were obtained using transthoracic echocardiography performed on Philips ultrasound systems (Philips Healthcare, Andover, MA, USA). Heart failure phenotypes were classified solely based on left ventricular ejection fraction, as additional clinical criteria, biomarker data, and standardized heart failure diagnoses were not consistently available in the dataset. Dyslipidemia was defined as a documented prior diagnosis of hyperlipidemia or active treatment with lipid-lowering therapy (e.g., statins) at the time of TAVR. Key laboratory values on admission and post-procedure (creatinine, hemoglobin, etc.), procedural details (valve type and size, degree of valve oversizing), and in-hospital outcomes (technical success, complications, need for permanent pacemaker, length of stay, readmissions, and mortality) were recorded. Atrial fibrillation and atrial flutter were not systematically extracted/recorded in this dataset and therefore were not analyzed. BMI was calculated as weight in kilograms divided by height in meters squared (kg/m^2^) and categorized a priori into <25 kg/m^2^, 25–29.9 kg/m^2^, and ≥30 kg/m^2^, corresponding to non-obese (lean/normal or underweight), overweight, and obese, respectively. Ethnicity was not recorded, as all patients were residents of the region and this study focused on BMI and clinical outcomes.

All patients who met the criteria underwent TAVR during the study period. No patient was excluded for extreme BMI explicitly, though extremely high BMI patients were rare in our sample. Estimated glomerular filtration rate (eGFR) was calculated for all patients using the CKD-EPI equation, indexed to a body surface area of 1.73 m^2^, and the same equation was applied uniformly to all patients. Weight-adjusted renal function formulas and therapeutic drug monitoring were not routinely performed and were not captured in the study dataset. Acute kidney injury (AKI), bleeding, vascular complications, stroke, and other peri-procedural endpoints were defined according to VARC-3 criteria as documented in the electronic health record. When granular staging (e.g., AKI stage) was not documented, AKI was noted if it was clinically diagnosed by the care team per hospital protocol (which aligns with KDIGO criteria) [12]. Multidetector computed tomography (MDCT) (Philips Healthcare, Best, The Netherlands) was used to measure the aortic annulus area and perimeter at mid-systole. Area- and perimeter-derived diameters were calculated as DA = 2√(A/π) and DP = P/π, respectively, according to established CT-based annular sizing methodology [13,14]. For balloon-expandable valves, percentage oversizing was calculated using the annular area, whereas for self-expandable valves the annular perimeter was used. Oversizing was computed using the following formula: oversizing (%) = [(nominal prosthesis dimension − CT annular dimension)/CT annular dimension] × 100, where the dimension was the area for balloon-expandable valves and the perimeter for self-expandable valves. In accordance with prior work on prosthesis oversizing in TAVR, valves were categorized as undersized (<0% oversizing), properly sized (0–<10%), moderately oversized (10–<20%), and excessively oversized (≥20%) [15,16].

The primary endpoint was the VARC-3 early safety composite during the index hospitalization. This composite included any of the following events (per VARC-3 definitions): all-cause death, major vascular complication, major bleeding, stroke, new permanent pacemaker implantation (for complete heart block), heart failure (HF) requiring urgent intervention, hypertensive emergency, acute kidney injury, or any valve-related reintervention before discharge. We additionally tracked in-hospital infections as an outcome of interest (not part of VARC-3), given our hypothesis about BMI and infection risk. “Infections” were defined as any significant infection during the hospitalization (such as pneumonia, sepsis, catheter-related bloodstream infection, or wound infection requiring treatment). These infectious events were analyzed separately from the VARC-3 composite. Frailty was not directly assessed in this study, and no standardized frailty measures (such as gait speed, Katz index, or sarcopenia markers) were collected. Secondary endpoints included the individual components of the composite, occurrence of moderate/severe paravalvular leak (PVL), cardiac tamponade or significant pericardial effusion, aortic regurgitation severity at discharge, total length of stay (LOS), 30-day readmission (and its causes), and all-cause mortality by 30 days and by 1 year.

We obtained follow-up clinical data for surviving patients through outpatient visits and records. New York Heart Association (NYHA) functional class was recorded at approximately 1 year and 2 years post-TAVR for those patients with available follow-up (about 40% of survivors at 1 year had a documented NYHA class). Follow-up echocardiograms were reviewed when available to note changes in LVEF. These longer-term data were collected to observe functional improvement over time.

Categorical variables are presented as counts and percentages, and continuous variables as mean ± standard deviation (SD) or median with range as appropriate. We used the Chi-square test or Fisher’s exact test for group comparisons of categorical variables, and Student’s *t*-test (for two groups) or one-way ANOVA (for three BMI groups) for approximately normally distributed continuous variables. If normality was violated (Kolmogorov–Smirnov test), we used non-parametric tests (Mann–Whitney U for two-group or Kruskal–Wallis for three-group comparisons). Univariate analyses were performed to identify factors associated with the occurrence of post-TAVR complications (the composite outcome). Univariate analyses (Chi-square/Fisher’s for categorical, *t*-test/ANOVA or Mann–Whitney/Kruskal–Wallis for continuous) were first performed to identify factors associated with the primary composite outcome. Variables significant at *p* < 0.10 in univariate analysis, as well as clinically important factors (age, sex, BMI category, and Society of Thoracic Surgeons (STS) risk category), were then considered in a multivariate logistic regression to determine independent predictors of in-hospital complications. We categorized STS predicted risk of mortality as low (<4%), moderate (4–8%), or high (>8%) for this analysis. A backward elimination approach was used (exit criterion *p* > 0.10) to refine the model. Adjusted odds ratios (ORs) with 95% confidence intervals (CIs) were calculated for each predictor. Model calibration was assessed with the Hosmer–Lemeshow goodness-of-fit test and discrimination by the c-statistic (area under the ROC curve). All tests were two-tailed, and *p* < 0.05 was considered statistically significant. Analyses were performed using SPSS version 26 (IBM Corp., Armonk, NY, USA).

This study was approved by the King Abdullah Medical City Biomedical Ethics Committee (IRB No. 24-1284), which is registered with the National Committee (KACST H-02-K-001) and adheres to ICH-GCP guidelines and OHRP regulations. Given the retrospective design, the requirement for individual informed consent was waived. The study had no external funding, and the authors declare no conflicts of interest. Patient data were de-identified and stored securely.

## 3. Results

A total of 197 patients underwent TAVR during the study period and met inclusion criteria. Table 1 presents the baseline characteristics of the patients stratified by BMI category.

Baseline Characteristics: The mean age of the cohort was 74.9 ± 8.8 years, with 51.3% of patients aged ≥ 75. Just over half (52.3%) were female. Notably, the BMI > 25 kg/m^2^ group was older on average than the higher-BMI groups (mean 75.5 years vs. 72.2 years in BMI ≥ 30; *p* < 0.001), and a greater proportion of patients with BMI < 25 were ≥75 years old (65.9% vs. 38.2% of the obese group; *p* = 0.003), Only three patients (1.5%) had BMI < 18.5 kg/m^2^, and none had BMI < 16.5 kg/m^2^, limiting inference about extreme underweight. More than three-quarters (77.7%) of patients were hypertensive and 57.9% were diabetic. Diabetes prevalence differed by BMI: It was significantly lower in the <25 kg/m^2^ group (38.6%) compared to overweight (62.5%) and obese (64.0%) patients (*p* = 0.013). Dyslipidemia was present in only 10.2% overall. Prior cardiac interventions included PCI in 18.8% and CABG in 6.1% of patients (no significant BMI-based differences). About 7% had peripheral vascular disease and 6% had ILD; 18.8% had chronic kidney disease (CKD, stage ≥ 3) and 5.6% were on dialysis for end-stage renal disease. History of stroke (old CVA) and TIA were relatively infrequent (10.7% and 2%, respectively). Patients in the overweight BMI category had a slightly higher incidence of prior TIA (≈9% vs. ~2–3% in others), but numbers were small (*p* = 0.67).

Risk scores indicated that 59.4% of patients were low-risk by EuroSCORE II (<4% predicted mortality) and the remainder moderate to high risk. BMI was not significantly associated with EuroSCORE II category (the distribution of low vs. higher EuroSCORE II was nearly identical across BMI groups, *p* ≈ 0.99). By STS score, 25% were low risk, 49% moderate, and 26% high risk. There was a significant association between BMI and STS risk category (*p* = 0.039): A smaller proportion of obese patients were in the high-STS group (15.7% vs. 36.4% of <25 BMI patients were high-risk by STS), and conversely, more obese patients fell into moderate risk. In other words, patients with lower BMI tended to have higher surgical risk profiles (likely reflecting comorbid frailty), whereas obese patients often had lower STS risk (consistent with their younger age). No other baseline characteristic differed significantly by BMI category. In summary, the <25 kg/m^2^ group was older and contained more high STS risk and fewer diabetics, whereas the obese group was younger, with more diabetics, but baseline comorbid burden was otherwise similar.

**Figure 1:**
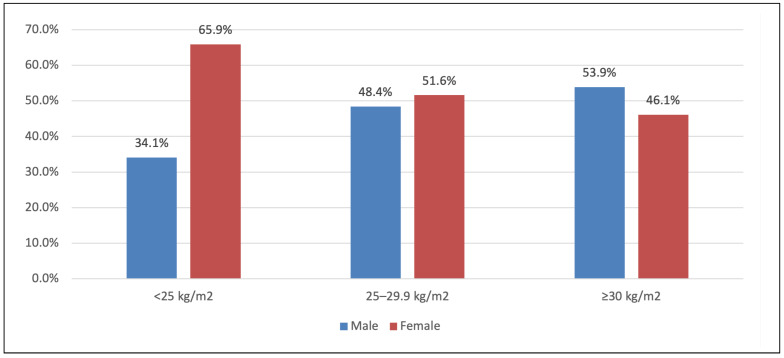
BMI level between males and females. BMI distribution by sex. Proportion of patients in each BMI category (<25 vs. ≥25 kg/m^2^) stratified by gender. (Females constituted 65.9% of the <25 BMI group vs. 34.1% males; this difference was not statistically significant).

Figure 1 illustrates the BMI distribution by sex. Females made up a higher proportion of the <25 kg/m^2^ group (65.9%) than of the obese group (46.1%). There was a trend toward women being more common in the low-BMI category, but overall BMI category distribution by sex was not statistically significant (*p* = 0.097).

**Figure 2:**
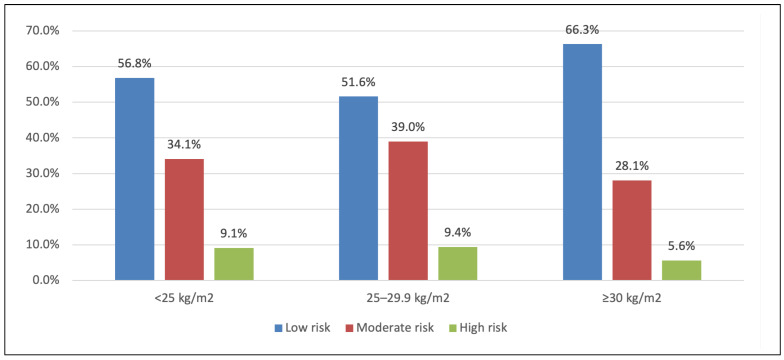
BMI level in relation to EuroSCORE II. BMI and EuroSCORE II risk categories. Percentage of patients classified as low, moderate, or high operative risk by EuroSCORE II, within each BMI category. (Obese patients (BMI ≥ 30) were slightly more likely to be low risk by EuroSCORE II, though differences were not significant, *p* = 0.99).

**Figure 3:**
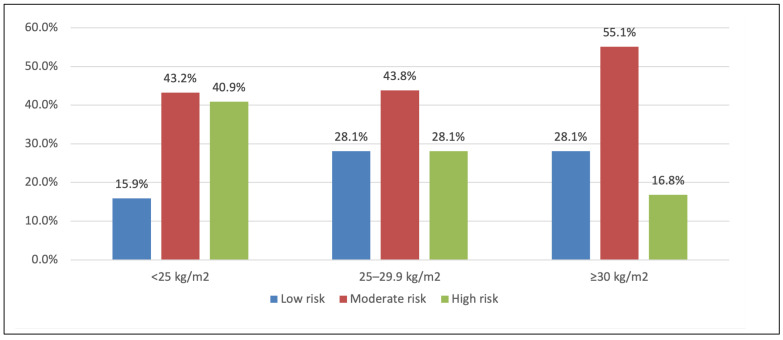
BMI level in relation to STS score. BMI and STS risk categories. Percentage of patients with low, moderate, or high STS-predicted risk of mortality, stratified by BMI category. (A greater proportion of obese patients were moderate risk by STS, while more normal BMI patients were high risk. Overall association *p* = 0.039).

Figure 2 and Figure 3 depict BMI categories in relation to operative risk strata (EuroSCORE II and STS). Patients with BMI ≥ 30 were slightly more likely to be in a low EuroSCORE II risk group (Figure 2), and more of the obese group fell into moderate STS risk (Figure 3), consistent with their age and comorbidity profile, but these trends did not reach significance in our cohort.

After TAVR, clinical status and anatomic characteristics were similar across BMI groups (Table 2). At baseline NYHA (available for ~60% of surviving patients), 26.4% of patients were in NYHA Class III and 26.4% in Class II, while only 4.6% reported no heart failure symptoms (Class I). These NYHA functional class distributions did not differ significantly by BMI level at any time point (all *p* > 0.5). Approximately 8.1% had experienced syncope prior to TAVR and 7.6% had a bicuspid aortic valve—neither differed by BMI (*p* = 0.79 and *p* = 0.12). Severe aortic valve calcification was present in 40.1% overall, and 14.2% had significant LVOT calcification, with no BMI-based differences (*p* = 0.48 and *p* = 0.80). The mean LVEF was 50.1% (±9.3); baseline LVEF was available in 137 patients (69.5%). Using LVEF thresholds, 15 (10.9%) met criteria for Heart Failure with Reduced Ejection Fraction (HFrEF) (LVEF < 40%), 17 (12.4%) for Heart Failure with Mid-range Ejection Fraction (HFmrEF) (LVEF 40–49%), and 105 (76.6%) for Heart Failure with Preserved Ejection Fraction (HFpEF) (LVEF ≥ 50%). Average aortic annulus area by CT was 408.2 ± 89.1 mm^2^ and mean annular perimeter was 73.5 ± 8.7 mm. These echocardiographic and anatomic measures were also similar among BMI groups (*p* > 0.1 for LVEF, annulus area, and perimeter). In summary, BMI showed no correlation with baseline valve morphology or early post-TAVR functional status.

Procedural Details and In-Hospital Complications: Nearly all patients (196, 99.5%) underwent TAVR via transfemoral approach, with only 1 case (0.5%) using subclavian access (Table 3). Technical success of the procedure was 99.0% (195 of 197 patients achieved device success). Balloon-expandable valves (Edwards SAPIEN series) were used in 74 patients (37.6%), while self-expandable valves (Medtronic CoreValve/Evolut series) were used in 123 patients (62.4%), with no significant differences across BMI categories (*p* = 0.162). Valve sizing relative to annulus was proper in ~35%, undersized in 22.8%, moderately oversized in 13.7%, and excessively oversized in 27.9%—these proportions were similar across BMI groups (*p* = 0.656). Procedural success rates were uniformly high across all BMI categories (98–100%, *p* = 1.00).

Regarding in-hospital outcomes (Table 3), the overall in-hospital all-cause mortality was 2.0% (4/197). Major stroke occurred in 3 patients (1.5%). Acute kidney injury was noted in 22 patients (11.2%). Major vascular complications occurred in 17 patients (8.6%). Major access site bleeding occurred in 22 patients (11.2%), all managed without long-term sequelae. New complete heart block requiring permanent pacemaker implantation occurred in 15 patients (7.6%). Aortic root rupture was rare (1 patient, 0.5%). Valve-in-valve implantation for acute device failure was needed in 2 patients (1.0%). Clinically significant PVL (moderate or severe) was detected in 43 patients (21.8%) before hospital discharge (most were moderate and managed conservatively). Table 3 details these complication rates by BMI category.

Importantly, BMI was not associated with the incidence of the primary composite outcome (the overall major complication rate did not differ by BMI category, *p* > 0.5 for the comparison). The only significant difference we observed was in the rate of in-hospital infections: Overweight patients had a higher infection incidence compared to both obese and normal-BMI patients (14.1% vs. 3.4% and 2.3%, respectively; *p* = 0.020). These infections included sepsis, pneumonia, or significant access-site infections requiring antibiotics. No other individual complication (stroke, AKI, bleeding, etc.) differed significantly among BMI groups (all *p* > 0.05).

In-Hospital Outcomes: Detailed outcome and treatment data are shown in Table 4. In-hospital all-cause mortality was 2.0% (4 patients). Mortality within 1-year post-procedure occurred for a total of 4 patients (2.0%). Beyond one year, 3 (1.5%) additional patients died. Approximately 94.5% of patients survived beyond 1 year post-TAVR. There was no statistically significant difference in mortality by BMI category (in-hospital or at 1 year, *p* = 0.44 and *p* = 0.23, respectively).

The readmission rate was 12.7% (25 patients). Among these 25 readmitted patients, the leading cause of readmission was heart HF exacerbation (9 cases, 36%), followed by acute coronary syndrome (ACS; 6 cases, 24%). There were 1 stroke (4% of readmissions), 2 AKI episodes (8%), 1 vascular complication (4%), 1 case of high-grade AV block (4%), and 5 non-cardiac readmissions (20%) such as infection or other issues. Overweight patients had the highest numerical readmission rate (15.6% vs. ~13.5% in obese and 6.8% in <25 BMI), but this was not statistically significant (*p* = 0.387). Causes of readmission did not differ significantly by BMI group (*p* = 0.28 for distribution of causes). In summary, BMI did not impact the likelihood or causes of readmission.

Permanent pacemaker implantation was required in 15 patients (7.6%) during the index hospitalization (all for complete AV block as noted). This rate did not vary by BMI (*p* = 1.00 in Table 3). A significant in-hospital hemoglobin drop (defined as >3 g/dL drop or requiring transfusion) occurred in 16 patients (8.1%), closely paralleling the “major bleeding” count, and likewise showed no BMI association (*p* = 0.495).

By the 1-year follow up post-TAVR, 30.5% were in class I, and by the second year 31.0% were in class I, reflecting general improvement post-TAVR compared to the baseline NYHA. Follow-up LVEF was available for 120 patients. The mean LVEF at follow-up was 53.8 ± 5.5%, compared with a baseline mean of 50.1 ± 9.3%. This modest improvement did not differ significantly across BMI categories (*p* = 0.223).

Laboratory indices stratified by BMI category are shown in Table 5. At admission, renal function was mildly impaired overall (mean serum creatinine 1.34 ± 1.22 mg/dL; eGFR 64.5 ± 25.5 mL/min/1.73 m^2^), with obese patients displaying numerically lower creatinine and higher eGFR than normal-weight and overweight patients both at baseline and post-TAVR; however, these differences did not reach statistical significance (all *p* > 0.09). BNP levels were markedly elevated in all groups without a consistent BMI-related trend. Admission and discharge hemoglobin values, platelet indices, admission glucose, electrolytes, HbA1c in diabetic patients, and troponin concentrations (baseline and peak post-TAVR) were broadly comparable across BMI categories (all *p* > 0.20). Overall, BMI was not associated with meaningful differences in baseline or post-procedural laboratory profiles.

The median total hospital length of stay (LOS) was 8 days (range 1–77, reflecting some prolonged rehospitalizations for complications). Post-TAVR LOS (from procedure to discharge) had a median of 2 days (range 1–63; many patients discharged on day 2 or 3 post-TAVR). Neither total LOS nor post-procedure LOS differed by BMI (*p* = 0.336 and *p* = 0.498, respectively). However, patients who experienced major complications had significantly longer stays (median 17.5 days vs. 6 days for those without complications; *p* < 0.001, see Table 6).

Regarding medications at discharge, 76.1% of patients were discharged on aspirin and 48.7% on clopidogrel (thus ~48% received DAPT). Only 2.5% received ticagrelor, as this was not routine for TAVR except in stented patients. Oral anticoagulants were prescribed in 26.9%. Beta-blockers were given to 63.5%. These treatment patterns were similar across BMI groups (all *p* > 0.1). Among the four patients (2.0%) with prior transient ischemic attack, two were discharged on dual antiplatelet therapy (aspirin plus clopidogrel) and two on clopidogrel monotherapy. None of these patients were discharged on oral anticoagulation.

In summary, BMI had no significant influence on in-hospital or 1-year outcomes in our cohort. Obese patients did not experience higher mortality or complication rates compared to non-obese patients, and likewise underweight/normal BMI patients had outcomes similar to others. The only notable difference—a higher infection rate in the overweight subgroup—did not translate into worse overall outcomes and may relate to confounding (e.g., more diabetics in that group).

Univariate Predictors of Complications: In univariate analysis to identify the factors influencing complications (Table 6), we found that complications were associated with increasing age (*p* = 0.006), those who were classified as high risk based on STS score (*p* = 0.020), those without clopidogrel prescription (*p* = 0.037), and those who had longer duration of hospital stay (*p* < 0.001). No significant differences were observed between complications in relation to gender, BMI level, diabetes, dyslipidemia, hypertension, prior PCI, chronic kidney disease (CKD), end-stage renal disease (ESRD), EuroSCORE II, LVOT calcification, valve categories, valve sizes and mismatch, aspirin, anticoagulant, beta-blockers, or aortic regurgitation (all *p* > 0.05).

Measuring the association between complications and laboratory parameters revealed that complications were associated with higher mean values of serum creatinine at admission (*p* = 0.009), while they were associated with lower mean values of eGFR at admission (*p* < 0.001) and hemoglobin at admission (*p* = 0.0330). In contrast, complications were associated with increasing mean values of glucose (*p* = 0.028) and initial troponin (*p* = 0.022). No significant differences were observed between complications in terms of LVEF, annulus area, perimeter, left ventricle ejection fraction (LVEF), BNP, platelets, MPV, sodium, potassium, or HbA1c (all *p* > 0.05) (Table 7).

On multivariate logistic regression (Table 8), age ≥ 75 years was associated with significantly higher odds of complications (adjusted OR 2.52, 95% CI 1.26–5.04; *p* = 0.009), and STS risk was also predictive: Patients in the moderate-risk category had ~3.7 times higher odds of complications than low-risk patients (OR ~3.75, 95% CI ~1.4–9.9; *p* = 0.008), and high-risk patients had ~2.3 times higher odds (OR ~2.26, 95% CI ~1.07–4.78; *p* = 0.033). Admission hemoglobin, glucose, and troponin did not reach statistical significance after adjustment (all *p* > 0.05). Although admission serum creatinine entered the model with a statistically significant odds ratio, the direction of effect was opposite to the univariate association (odds ratio < 1 despite higher creatinine among patients with complications); therefore, this parameter was not considered a robust independent predictor. BMI category was not independently associated with in-hospital complications. The final model showed acceptable discrimination (c-statistic 0.72) and good calibration (Hosmer–Lemeshow *p* = 0.88).

## 4. Discussion

In this single-center Middle Eastern TAVR cohort, BMI was not an independent predictor of in-hospital major adverse outcomes. The incidence of the VARC-3 composite safety endpoint and in-hospital mortality were similar across BMI categories. The only significant BMI-related difference was a higher rate of post-procedural infections in the overweight group compared to both normal-weight and obese patients (14.1% vs. 2.3% and 3.4%, respectively; *p* = 0.020). Apart from this finding, our hypothesis that BMI would not adversely impact short-term TAVR outcomes was confirmed. Technical success exceeded 99%, and overall in-hospital mortality was low (2.0%). These results indicate that patients with elevated BMI can undergo TAVR without excess peri-procedural risk, consistent with the “obesity paradox” phenomenon described in other populations [4,5,6,7,17].

In a large registry analysis (>30,000 TAVR patients), Sharma et al. [4] reported that, compared to normal BMI, underweight status was associated with higher 30-day and 1-year mortality, whereas overweight and class I–II obesity were associated with significantly lower adjusted 1-year mortality (hazard ratios ~0.80–0.88). Similarly, an international TAVR cohort study by van Nieuwkerk et al. [5] found no increase in 30-day mortality in overweight or obese patients versus those with normal BMI, with underweight patients instead showing the highest mortality rates.

It should be noted that the apparent protective effect of higher BMI may not extend to the end of the spectrum: Several analyses suggest that severe obesity (BMI ≥ 40 kg/m^2^) attenuates or abolishes any outcome advantage and may be associated with increased procedural risk and access-site complications [6,7]. Moreover, not all populations demonstrate an obesity paradox to the same extent. A recent nationwide TAVR registry from China (National Transcatheter Valve Therapeutics Registry) found no clear survival benefit in overweight/obese patients and higher complication rates at very high or very low BMI, likely reflecting a lower overall BMI distribution and differing clinical characteristics in East Asian cohorts [18]. Overall, our results are in line with the majority of Western TAVR studies reporting neutral or positive short-term outcomes in higher-BMI patients, and our study extends those observations to an understudied Middle Eastern population [4,5,6,7,17,18].

In contrast, very low BMI is frequently a surrogate for sarcopenia, cachexia, or chronic illness, and patients with low BMI are often more frail and thus more vulnerable to procedural stress [8,17,18,19,20,21]. In the FRAILTY-AVR study, frailty more than tripled the odds of death or poor functional status at one year after valve intervention [20]. Similarly, Dautzenberg et al. showed that frailty in older TAVR candidates was associated with higher rates of post-procedural complications and mortality [19].

In our cohort, patients with BMI < 25 were significantly older on average than those with obesity (mean 75.5 vs. 72.2 years), and a greater proportion of normal-BMI patients were ≥ 75 years old. Normal-weight patients were also more likely to be in a high surgical risk category than obese patients. These differences suggest that the “leaner” patients in our study carried a higher burden of frailty and comorbidities, which likely contributed to any excess complications in that group. Consistent with this, we found that age ≥ 75 was associated with over twice the odds of in-hospital complications (adjusted OR 2.52), and a higher STS risk score was a strong independent predictor as well (~3.7-fold higher odds for moderate vs. low risk). These observations align with prior work demonstrating that frailty indices add prognostic value beyond conventional risk scores in TAVR [19,20]. Although renal dysfunction, reflected by higher serum creatinine and lower estimated glomerular filtration rate, was significantly associated with in-hospital complications on univariate analysis, it did not retain independent significance in the final multivariable model after adjustment for age and STS risk. This likely reflects the close relationship between renal impairment, global frailty, and overall operative risk burden, which are already captured by established surgical risk scores. Therefore, renal function appears to act as a marker of vulnerability rather than an independent mechanistic driver of early adverse outcomes in this cohort.

One notable observation in our study was the higher incidence of in-hospital infections in overweight patients. Prior TAVR series have not highlighted infection risk as varying systematically by BMI, and overall post-TAVR infection rates are low given the minimally invasive nature of the procedure [4,5,6,7,10,11]. In our cohort, however, the overweight group had a significantly higher prevalence of diabetes than the normal-BMI group (62.5% vs. 38.6%), which could predispose to infections such as pneumonia or sepsis. This correlation should be viewed as hypothesis-generating rather than confirmed.

It is possible that the combination of older age and metabolic comorbidities in the overweight subgroup contributed to this finding. We interpret this result with caution; the absolute number of infection events was relatively small, and we examined multiple endpoints, raising the possibility of a chance finding. Nonetheless, this signal underlines the importance of vigilant perioperative management in patients with elevated BMI and metabolic risk factors. Future studies should explore this association further to determine whether it is reproducible or a spurious observation [6,7,18].

From a clinical standpoint, our results suggest that BMI alone should not be a major factor in TAVR candidacy or peri-procedural risk stratification. Obese patients (BMI ≥ 30 kg/m^2^) in our study had short-term outcomes equivalent to those of normal-weight patients, indicating that concerns about performing TAVR in high-BMI individuals may be overstated [4,5,6,7,17,18]. This is particularly relevant in regions like the Middle East, which have among the highest obesity prevalence globally [9]. Our data provide reassurance that TAVR can be safely and effectively performed across a wide BMI spectrum, and that patients with obesity should not be declined or deemed high risk for TAVR purely due to body size.

Instead, greater emphasis should be placed on comprehensive geriatric assessment and quantification of frailty, since those factors more directly influence outcomes [19,20]. In practice, this means that a fit obese patient should be considered a good TAVR candidate if otherwise indicated, whereas a frail normal-weight patient may have higher risks despite a “healthy” BMI. Risk models might therefore require refinement to incorporate frailty indices or markers of nutritional status rather than relying on BMI alone [17,19,20].

Additionally, we underscore the need to optimize modifiable comorbidities common in higher-BMI patients (such as diabetes, as noted above) to further improve outcomes. Ensuring meticulous technique for vascular access is important as well. Overall, the clinical message is that an elevated BMI is not a contraindication to TAVR; patient selection should focus on the overall risk profile and technical considerations, not weight alone [4,5,6,7,17,18,19,20].

### Strengths and Limitations

This study adds to the literature by providing data on BMI and TAVR outcomes in a non-Western population, using contemporary devices and standardized endpoint definitions. All events were adjudicated per VARC-3 criteria, lending rigor and comparability to our findings. Technical success was defined according to VARC-3 and exceeded 99%, consistent with recent validation work showing that achieving VARC-3 technical success is strongly associated with improved 1-year cardiovascular outcomes.

The cohort included patients across the spectrum of surgical risk (from low to high STS risk), enhancing generalizability to real-world TAVR practice. However, several limitations merit consideration. First, the sample size (*n* = 197) is modest, and the number of events for certain outcomes (e.g., death or stroke) was small, which may limit our power to detect subtle differences between BMI groups. In particular, the extremes of BMI were underrepresented; we had very few patients with BMI < 18.5 or ≥40 (as is common in many TAVR series). Therefore, generalizability to extreme BMI categories (<18.5 or ≥40 kg/m^2^) is limited. Our analysis lumped together all underweight/normal-weight patients and all obese classes. Because NYHA class and echocardiographic follow-up were available for only a subset of survivors, functional outcome comparisons across BMI groups are underpowered and should be interpreted cautiously.

Second, this was an observational single-center study, and unmeasured confounders (e.g., detailed frailty metrics or nutritional status markers) could influence both BMI and outcomes. We did not collect standardized frailty tools such as the Katz Activities of Daily Living index, gait speed testing, CT-based sarcopenia measurements, or nutritional biomarkers, which are strongly associated with outcomes after TAVR; thus, residual confounding by frailty could mask or mimic an “obesity paradox.”

Third, BMI itself is an imperfect measure of adiposity and health; it does not distinguish between fat and muscle mass or account for fat distribution. Emerging data suggest that CT-based assessments of visceral and subcutaneous fat and skeletal muscle may better predict post-TAVR outcomes than BMI alone. Some obese patients may have high muscle mass and good fitness (so-called “metabolically healthy” obesity), while some normal-BMI patients are metabolically unhealthy or sarcopenic (harboring “hidden” frailty), complicating the interpretation of BMI outcome associations.

Fourth, frailty was not assessed using standardized measures (e.g., gait speed, ADL indices, or sarcopenia metrics). Given the known relationship between frailty, BMI, and TAVR outcomes, residual confounding by unmeasured frailty cannot be excluded.

Fifth, atrial fibrillation and flutter were not systematically captured in the dataset; therefore, we could not assess their prevalence or potential impact on symptoms, anticoagulation decisions, or outcomes.

Sixth, follow-up was incomplete for functional and echocardiographic outcomes, with NYHA class available for approximately 40% of survivors, and incomplete longitudinal follow-up potentially leading to underestimation of late functional outcomes. In addition, 30-day readmissions were ascertained from our institutional electronic medical records only; unplanned admissions to other hospitals were not systematically captured, which may have led to underestimation of true readmission rates.

Additionally, our finding of increased infections in the overweight group should be interpreted cautiously. We identified infections from clinical records but did not adjudicate them with an external committee; minor or subclinical infections might have been missed, and the statistical significance could be influenced by multiple comparisons. Finally, our analysis focused on in-hospital outcomes (with data on 30-day events); we did not have complete longitudinal follow-up beyond one year. It remains possible that BMI could influence longer-term outcomes such as late mortality or structural valve degeneration, which were beyond the scope of this study.

## 5. Conclusions

In conclusion, among patients undergoing TAVR at our center, BMI was not associated with an increase in in-hospital complications or mortality. Patients with obesity had short-term outcomes comparable to those with normal body weight, reinforcing that TAVR can be offered to obese patients without expecting higher peri-procedural risk. Instead, advanced age and higher operative risk scores were the key drivers of adverse outcomes in our cohort, underscoring the importance of frailty and overall health status in determining TAVR prognosis.

Our study, the first to examine BMI effects on TAVR in a Middle Eastern population, suggests that the “obesity paradox” observed in Western cohorts is also evident in this region, likely due to confounding by patient frailty rather than a true protective effect of adiposity. Larger, multicenter studies in diverse populations are warranted to confirm these results and to further elucidate the interactions between body composition, patient frailty, and TAVR outcomes. Such efforts will help refine risk stratification and ensure that we appropriately select patients for this life-saving procedure, regardless of body size.

## Figures and Tables

**Table 1:** Baseline characteristics by BMI category (*n* = 197).

Study Data	Overall N (%) (*n* = 197)	BMI Level			*p*-Value ^§^
		<25 kg/m^2^ N (%) (*n* = 44)	25–29.9 kg/m^2^ N (%) (*n* = 64)	≥30 kg/m^2^ N (%) (*n* = 89)	
Age in years (mean ± SD) ^‡^	74.9 ± 8.75	75.5 ± 10.2	76.1 ± 7.79	72.2 ± 7.82	<0.001 **
• <75 years	96 (48.7%)	15 (34.1%)	26 (40.6%)	55 (61.8%)	0.003 ** ^†^
• ≥75 years	101 (51.3%)	29 (65.9%)	38 (59.4%)	34 (38.2%)	
Gender					
• Male	94 (47.7%)	15 (34.1%)	31 (48.4%)	48 (53.9%)	0.097 ^†^
• Female	103 (52.3%)	29 (65.9%)	33 (51.6%)	41 (46.1%)	
Diabetes mellitus	114 (57.9%)	17 (38.6%)	40 (62.5%)	57 (64.0%)	0.013 ** ^†^
DLP	20 (10.2%)	8 (18.2%)	6 (9.4%)	6 (6.7%)	0.117 ^†^
HTN	153 (77.7%)	29 (65.9%)	52 (81.2%)	72 (80.9%)	0.105 ^†^
Prior PCI	37 (18.8%)	11 (25.0%)	12 (18.8%)	14 (15.7%)	0.436 ^†^
Prior CABG	12 (6.1%)	3 (6.8%)	4 (6.3%)	5 (5.6%)	1.000
Prior valve surgery	4 (2.0%)	1 (2.3%)	1 (1.6%)	2 (2.2%)	1.000
PVD	14 (7.1%)	3 (6.8%)	4 (6.3%)	7 (7.9%)	0.938
ILD	12 (6.1%)	3 (6.8%)	3 (04.7%)	6 (6.7%)	0.862
CKD	37 (18.8%)	7 (15.9%)	12 (18.8%)	18 (20.2%)	0.835
ESRD	11 (5.6%)	03 (6.8%)	06 (9.4%)	02 (2.2%)	0.120
Prior TIA	4 (2.0%)	0 (0%)	04 (6.3%)	0 (0%)	0.013 **
Old CVA	21 (10.7%)	05 (11.4%)	12 (18.8%)	04 (4.5%)	0.018 **
EuroSCORE II					
• Low risk	117 (59.4%)	25 (56.8%)	33 (51.6%)	59 (66.3%)	0.423
• Moderate risk	65 (33.0%)	15 (34.1%)	25 (39%)	25 (28.1%)	
• High risk	15 (7.6%)	4 (9.1%)	6 (9.4%)	5 (5.6%)	
STS score					
• Low risk	50 (25.4%)	07 (15.9%)	18 (28.1%)	25 (28.1%)	0.039 ** ^†^
• Moderate risk	96 (48.7%)	19 (43.2%)	28 (43.8%)	49 (55.1%)	
• High risk	51 (25.9%)	18 (40.9%)	18 (28.1%)	15 (16.8%)	

^§^ *p*-value has been calculated using Fisher’s exact test. ^†^ *p*-value has been calculated using Chi-square test. ^‡^ *p*-value has been calculated using one-way ANOVA test. ** Significant at *p* < 0.05 level. EuroSCORE II and STS risk categories defined as low < 4%, moderate 4–8%, high > 8% predicted mortality. (STS: Society of Thoracic Surgeons risk score; EuroSCORE II: European System for Cardiac Operative Risk Evaluation II.) Abbreviations: SD = standard deviation; PCI = percutaneous coronary intervention; CABG = coronary artery bypass grafting; PVD = peripheral vascular disease; ILD = interstitial lung disease; CKD = chronic kidney disease; TIA = transient ischemic attack; CVA = cerebrovascular accident (stroke); DLP = dyslipidemia.

**Table 2:** Functional status and anatomic characteristics (by BMI category).

Variables	Overall N (%) (*n* = 197)	BMI Level			*p*-Value ^§^
		<25 kg/m^2^ N (%) (*n* = 44)	25–29.9 kg/m^2^ N (%) (*n* = 64)	≥30 kg/m^2^ N (%) (*n* = 89)	
NYHA at admission ^†^					0.842
• Class I	9 (4.6%)	1 (2.3%)	4 (6.3%)	4 (4.5%)	
• Class II	52 (26.4%)	12 (27.3%)	17 (26.6%)	23 (25.8%)	
• Class III	52 (26.4%)	9 (20.5%)	17 (26.6%)	26 (29.2%)	
• Class IV	2 (1.0%)	1 (2.3%)	1 (1.6%)	0 (0%)	
History of syncope	16 (8.1%)	4 (9.1%)	6 (9.4%)	6 (6.7%)	0.793
Bicuspid aortic valve	15 (7.6%)	6 (13.6%)	2 (3.1%)	7 (7.9%)	0.125
Severe aortic valve calcification	79 (40.1%)	20 (45.5%)	22 (34.4%)	37 (41.6%)	0.477 ^†^
LVOT calcification	28 (14.2%)	7 (15.9%)	10 (15.6%)	11 (12.4%)	0.795 ^†^
LVEF (%) (mean ± SD) ^‡^	50.1 ± 9.32	48.0 ± 11.2	49.5 ± 10.7	51.5 ± 7.02	0.237
Annulus area (mean ± SD) ^‡^	408.2 ± 89.1	415.9 ± 88.0	389.5 ± 90.5	416.9 ± 88.0	0.198
Perimeter (mean ± SD) ^‡^	73.5 ± 8.68	74.7 ± 7.23	71.5 ± 7.78	74.2 ± 9.73	0.167
Pericardial effusion	12 (6.1%)	2 (4.5%)	6 (9.4%)	4 (4.5%)	0.437
Aortic regurgitation					
None	94 (47.7%)	24 (54.5%)	28 (43.8%)	42 (47.2%)	0.196 ^†^
Mild	61 (31.0%)	11 (25.0%)	17 (26.6%)	33 (37.1%)	
Moderate to severe	42 (21.3%)	9 (20.5%)	19 (29.7%)	14 (15.7%)	

^§^ *p*-value has been calculated using Fisher’s exact test. ^†^ *p*-value has been calculated using Chi-square test. ^‡^ *p*-value has been calculated using one-way ANOVA test. Abbreviations: NYHA = New York Heart Association functional class; AV = aortic valve; LVOT = left ventricular outflow tract; LVEF = left ventricular ejection fraction.

**Table 3:** Procedural details and in-hospital complications by BMI.

Variables	Overall N (%) (*n* = 197)	BMI Level			*p*-Value ^§^
		<25 kg/m^2^ N (%) *(n* = 44)	25–29.9 kg/m^2^ N (%) (*n* = 64)	≥30 kg/m^2^ N (%) (*n* = 89)	
Valve categories					
• Balloon expandable Valve	74 (37.6%)	21 (47.7%)	19 (29.7%)	34 (38.2%)	0.162 ^†^
• Self-expandable Valve	123 (62.4%)	23 (52.3%)	45 (70.3%)	55 (61.8%)	
Valve sizes and mismatch					
• Undersized	45 (22.8%)	9 (20.5%)	14 (21.9%)	22 (24.7%)	0.656 ^†^
• Properly sized	70 (35.5%)	21 (47.7%)	21 (32.8%)	28 (31.5%)	
• Moderately oversized	27 (13.7%)	5 (11.4%)	10 (15.6%)	12 (13.5%)	
• Excessively oversized	55 (27.9%)	9 (20.5%)	19 (29.7%)	27 (30.3%)	
Procedural approach					
• Femoral	196 (99.5%)	44 (100%)	63 (98.4%)	89 (100%)	0.548
• Subclavian	1 (0.50%)	0	1 (1.6%)	0	
Procedural success	195 (99.0%)	44 (100%)	63 (98.4%)	88 (98.9%)	1.000
Stroke (major ischemic or hemorrhagic)	3 (1.5%)	0	1 (1.6%)	2 (02.2%)	1.000
AKI	22 (11.2%)	5 (11.4%)	9 (14.1%)	8 (9.0%)	0.616 ^†^
Major vascular complication	17 (8.6%)	5 (11.4%)	8 (12.5%)	4 (4.5%)	0.168
Major access site bleeding	22 (11.2%)	8 (18.2%)	8 (12.5%)	6 (6.7%)	0.132 ^†^
New complete heart block (CHB)	15 (7.6%)	3 (6.8%)	5 (7.8%)	7 (7.9%)	
Aortic rupture	1 (0.50%)	1 (2.3%)	0	0	0.223
Emergency valve-in-valve implant	2 (1.0%)	0	1 (1.6%)	01 (1.1%)	1.000
Post-TAVR infection	13 (6.6%)	1 (2.3%)	9 (14.1%)	3 (3.4%)	0.020 **
Moderate or severe PVL	43 (21.8%)	08 (18.2%)	14 (21.9%)	21 (23.6%)	0.777 ^†^

^§^ *p*-value has been calculated using Fisher’s exact test. ^†^ *p*-value has been calculated using Chi-square test. ** Significant at *p* < 0.05 level. Abbreviations: PPM = permanent pacemaker (for CHB); PVL = paravalvular leak; CHB = complete heart block.

**Table 4:** In-hospital and 30-day outcomes, readmissions, and discharge medications.

Variables	Overall N (%) (*n* = 197)	BMI Level			*p*-Value ^§^
		<25 kg/m^2^ N (%) (*n* = 44)	25–29.9 kg/m^2^ N (%) (*n* = 64)	≥30 kg/m^2^ N (%) (*n* = 89)	
In-hospital mortality	4 (2.0%)	2 (4.5%)	1 (1.6%)	1 (1.1%)	0.440
Mortality within 1 year	4 (2.0%)	2 (4.5%)	2 (3.1%)	0 (0%)	0.107
Mortality > 1 year	3 (1.5%)	1 (2.3%)	2 (3.1%)	0 (0%)	0.230
Readmission	25 (12.7%)	3 (6.8%)	10 (15.6%)	12 (13.5%)	0.387
Cause of readmission (*n* = 25)					
• HF exacerbation	9 (36.0%)	0 (0%)	4 (40.0%)	5 (41.7%)	0.280
• ACS	6 (24.0%)	2 (66.7%)	2 (20.0%)	2 (16.7%)	
• Stroke	1 (4.0%)	0 (0%)	0 (0%)	1 (8.3%)	
• AKI	2 (08.0%)	1 (33.3%)	1 (10.0%)	0 (0%)	
• Vascular complication	1 (04.0%)	0 (0%)	1 (10.0%)	0 (0%)	
• High-grade AV block	1 (4.0%)	0 (0%)	1 (10.0%)	0 (0%)	
• Non-cardiac	5 (20.0%)	0 (0%)	1 (10.0%)	4 (33.3%)	
New permanent pacemaker (in-hospital)	15 (7.6%)	3 (6.8%)	05 (7.8%)	7 (7.9%)	1.000
Hemoglobin drop > 3 g/dL or transfusion	16 (8.1%)	4 (9.1%)	03 (4.7%)	9 (10.1%)	0.495
NYHA at 1-year follow up					
Class I	60 (30.5%)	10 (22.7%)	18 (28.1%)	32 (36.0%)	0.092
Class II	15 (7.6%)	1 (2.3%)	4 (16.3%)	10 (11.2%)	
Class III	03 (1.5%)	2 (4.5%)	0 (0%)	1 (1.1%)	
NYHA at 2-year follow up					
Class I	61 (31.0%)	13 (29.5%)	19 (29.7%)	29 (32.6%)	0.497
Class II	09 (04.6%)	0 (0%)	3 (4.7%)	6 (6.7%)	
LVEF at 1-year follow-up (%)	53.8 ± 5.46 *(n* = 120)	55.1 ± 4.09	52.3 ± 7.37	54.6 ± 3.38	0.223
LOS in days, median (min-max) ^‡^	8 (0–77)	8 (2–38)	9.5 (0–77)	6 (0–60)	0.336
LOS post-TAVI in days, median (min-max) ^‡^	2 (0–63)	3 (1–37)	3 (0–63)	2 (1–48)	0.498
Aspirin on discharge	150 (76.1%)	36 (81.8%)	49 (76.6%)	65 (73.0%)	0.533 ^†^
Clopidogrel on discharge	96 (48.7%)	19 (43.2%)	34 (53.1%)	43 (48.3%)	0.594 ^†^
Ticagrelor on discharge	5 (2.5%)	2 (4.5%)	2 (3.1%)	1 (1.1%)	0.518
Anticoagulant on discharge	53 (26.9%)	8 (18.2%)	16 (25.0%)	29 (32.6%)	0.194 ^†^
Beta-blocker on discharge	125 (63.5%)	29 (65.9%)	40 (62.5%)	56 (62.9%)	0.928 ^†^

^§^ *p*-value has been calculated using Fisher’s exact test. ^†^ *p*-value has been calculated using Chi-square test. ^‡^ *p*-value has been calculated using Kruskal–Wallis test. (Readmission causes add to 25; each percentage is of readmitted patients. Medication percentages of those discharged alive). Abbreviations: AV = atrioventricular; LOS = length of stay; HF = heart failure; ACS = acute coronary syndrome; EF = ejection fraction.

**Table 5:** Laboratory values by BMI category.

Laboratory Variable	Overall Mean ± SD (*n* = 197)	BMI Level			*p*-Value ^‡^
		<25 kg/m^2^ Mean ± SD (*n* = 44)	25–29.9 Mean ± SD (*n* = 64)	≥30 kg/m^2^ Mean ± SD (*n* = 89)	
Serum creatinine-admission (mg/dL)	1.34 ± 1.22	1.51 ± 1.58	1.50 ± 1.26	1.14 ± 0.94	0.130
eGFR—admission (mL/min/1.73 m^2^)	64.5 ± 25.5	64.0 ± 27.1	59.4 ± 27.5	68.1 ± 22.8	0.137
Serum creatinine—post-TAVR (mg/dL)	1.41 ± 1.45	1.64 ± 2.10	1.61 ± 1.46	1.16 ± 0.96	0.099
eGFR-post-TAVR (mL/min/1.73 m^2^)	66.2 ± 28.1	65.5 ± 29.0	60.5 ± 30.3	70.4 ± 25.5	0.115
BNP (pg/mL)—admission	1315 ± 2202	1209 ± 1230	1710 ± 3409	1115 ± 1509	0.602
Hemoglobin—admission (g/dL)	11.8 ± 1.97	11.7 ± 2.15	11.4 ± 1.81	12.0 ± 1.97	0.148
Hemoglobin-discharge (g/dL)	11.0 ± 1.84	11.1 ± 2.19	10.7 ± 1.68	11.2 ± 1.73	0.353
Platelet count (×10^9^/L)—adm	233.3 ± 80.7	229.9 ± 78.4	219.9 ± 84.5	244.4 ± 78.5	0.203
Mean platelet volume (fL)	10.8 ± 1.11	10.9 ± 1.21	10.9 ± 1.08	10.8 ± 1.09	0.836
Glucose—admission (mg/dL)	155.2 ± 87.2	131.6 ± 45.3	188.5 ± 142	151.3 ± 55.3	0.282
Sodium—admission (mmol/L)	136.8 ± 4.13	136.9 ± 4.31	136.5 ± 4.13	136.9 ± 4.08	0.839
Potassium—admission (mmol/L)	4.18 ± 0.53	4.19 ± 0.55	4.13 ± 0.44	4.21 ± 0.59	0.681
HbA1c (%)—if diabetic	6.61 ± 1.67 (*n* = 114)	6.57 ± 2.10	6.49 ± 1.45	6.71 ± 1.59	0.818
Troponin (baseline, ng/mL)	0.47 ± 1.42	0.31 ± 0.47	0.59 ± 1.77	0.47 ± 1.42	0.638
Troponin (peak post-TAVR, ng/mL)	2.68 ± 12.3	3.75 ± 18.0	3.86 ± 14.2	1.11 ± 2.18	0.430

^‡^ *p*-value has been calculated using one-way ANOVA *t*-test. Abbreviations: eGFR = estimated glomerular filtration rate; BNP = B-type natriuretic peptide; HbA1c = hemoglobin A1c.

**Table 6:** Univariate analysis—factors associated with in-hospital complications (*n* = 197).

Factor	Complication		*p*-Value ^§^
	Yes N (%) (*n* = 50)	No N (%) (*n* = 147)	
Age group			
• <75 years	16 (32.0%)	80 (54.4%)	0.006 ** ^†^
• ≥75 years	34 (68.0%)	67 (45.6%)	
Gender			
• Male	26 (52.0%)	68 (46.3%)	0.483
• Female	24 (48.0%)	79 (53.7%)	
BMI level			
• <25 kg/m^2^	12 (24.0%)	32 (21.8%)	0.479
• 25–29.9 kg/m^2^	19 (38.0%)	45 (30.6%)	
• ≥30 kg/m^2^	19 (38.0%)	70 (47.6%)	
Diabetes mellitus	25 (50.0%)	89 (60.5%)	0.192
DLP	05 (10.0%)	15 (10.2%)	0.967
Hypertension	39 (78.0%)	114 (77.6%)	0.948
Prior PCI	9 (18.0%)	28 (19.0%)	0.870
CKD	13 (26.0%)	24 (16.3%)	0.130
ESRD	04 (08.0%)	07 (04.8%)	0.389
EuroSCORE II			
• Low risk	23 (46.0%)	94 (63.9%)	0.068
• Moderate risk	21 (42.0%)	44 (29.9%)	
• High risk	6 (12.0%)	09 (6.1%)	
STS score			
• Low risk	08 (16.0%)	42 (28.6%)	0.020 **
• Moderate risk	22 (44.0%)	74 (50.3%)	
• High risk	20 (40.0%)	31 (21.1%)	
Severe aortic valve calcification	20 (40.0%)	59 (40.1%)	0.986
LVOT calcification	08 (16.0%)	20 (13.6%)	0.675
Valve categories			
• Balloon-expandable valve	20 (40.0%)	54 (36.7%)	0.680
• Self-expandable valve	30 (60.0%)	93 (63.3%)	
Valve sizes and mismatch			
• Undersized	12 (24.0%)	33 (22.4%)	0.993
• Properly sized	17 (34.0%)	53 (36.1%)	
• Moderately oversized	07 (14.0%)	20 (13.6%)	
• Excessively oversized	14 (28.0%)	41 (27.9%)	
On aspirin			
• No	16 (32.0%)	31 (21.1%)	0.118
• Yes	34 (68.0%)	116 (78.9%)	
On clopidogrel			
• No	32 (64.0%)	69 (46.9%)	0.037 **
• Yes	18 (36.0%)	78 (53.1%)	
On anticoagulant			
• No	36 (72.0%)	108 (73.5%)	0.840
• Yes	14 (28.0%)	39 (26.5%)	
On beta-blockers			
• No	22 (44.0%)	50 (34.0%)	0.205
• Yes	28 (56.0%)	97 (66.0%)	
Aortic regurgitation			
• None	17 (34.0%)	77 (52.4%)	0.077
• Mild	19 (38.0%)	42 (28.6%)	
• Moderate-severe	14 (28.0%)	28 (19.0%)	
LOS in days (m ± SD) ^‡^	17.5 (2–77)	6 (0–68)	<0.001 **

^§^ *p*-value has been calculated using Chi-square test. ^‡^ *p*-value has been calculated using Mann–Whitney test. ^†^ *p*-value has been calculated using Chi-square test. ** Significant at *p* < 0.05 level. (“In-hospital death” is part of the composite outcome and thus trivially associated; length of stay is a consequence of complications). Abbreviations: AV = aortic valve; LVOT = left ventricular outflow tract; PCI = percutaneous coronary intervention; STS = Society of Thoracic Surgeons score; EuroSCORE II = European System for Cardiac Operative Risk Evaluation II.

**Table 7:** Association of baseline lab values with complications.

Laboratory Variable	Complications		*p*-Value ^§^
	Yes M ± SD (*n* = 50)	No M ± SD (*n* = 147)	
LVEF (%)	48.9 ± 10.7	50.6 ± 8.68	0.324
Annulus area (mm^2^)	393.3 ± 88.6	413.3 ± 89.1	0.210
Annulus perimeter (mm)	72.2 ± 7.62	73.9 ± 8.99	0.311
Creatinine—adm (mg/dL)	1.74 ± 1.50	1.20 ± 1.08	0.009 **
eGFR—adm (mL/min)	50.5 ± 22.7	69.3 ± 24.7	<0.001 **
BNP (pg/mL)—adm	1430.6 ± 1419.9	1247.7 ± 2565.9	0.735
Hemoglobin—adm (g/dL)	11.2 ± 1.89	11.9 ± 1.97	0.033 **
Platelet count (×10^9^/L)	232.8 ± 97.5	233.5 ± 74.7	0.958
MPV (fL)	10.9 ± 1.25	10.8 ± 1.07	0.428
Glucose—adm (mg/dL)	234.2 ± 177.4	143.6 ± 62.1	0.028 **
Sodium—adm (mmol/L)	136.5 ± 4.28	136.9 ± 4.09	0.634
Potassium—adm (mmol/L)	4.26 ± 0.69	4.16 ± 0.47	0.247
HbA1c (%)—if diabetic	6.10 ± 1.41	6.77 ± 1.71	0.073
Troponin—adm (ng/mL)	0.90 ± 2.02	0.32 ± 1.11	0.022 **

^§^ *p*-value has been calculated using one-way ANOVA *t*-test. ** Significant at *p* < 0.05 level. (Laboratory data available for ≥95% of patients; diabetic subset for HbA1c given). Abbreviations: adm = admission; MPV = mean platelet volume.

**Table 8:** Multivariate logistic regression for independent predictors of in-hospital complications (*n* = 197).

Factor	AOR	95% CI	*p*-Value
Age group			
• <75 years	Ref		
• ≥75 years	2.518	1.259–5.035	0.009 **
STS score			
• Low risk	Ref		
• Moderate risk	3.749	1.422–9.883	0.008 **
• High risk	2.260	1.069–4.782	0.033 **
Hemoglobin—adm (g/dL)	1.201	0.995–1.450	0.056
Glucose—adm (mg/dL)	0.988	0.977–1.000	0.059
Troponin—adm (ng/mL)	0.780	0.608–1.002	0.052

Adjusted for gender, diabetes, DLP, and HTN. ** Significant at *p* < 0.05 level. (Model adjusted for sex, diabetes, dyslipidemia, hypertension as well—none of those were significant; they are not shown for brevity). Abbreviations: OR = odds ratio; CI = confidence interval; STS = Society of Thoracic Surgeons risk category. Model c-statistic = 0.72; Hosmer–Lemeshow *p* = 0.88. *p* < 0.05 indicates statistical significance.

## Data Availability

The data supporting the findings of this study are not publicly available due to institutional privacy regulations and ethical restrictions related to patient confidentiality. De-identified data may be made available by the corresponding author upon reasonable request and with appropriate institutional approval.

## Abbreviations

The following abbreviations are used in this manuscript:

AKI	Acute kidney injury
ANOVA	Analysis of variance
AR	Aortic regurgitation
AS	Aortic stenosis
AVB	Atrioventricular block
BMI	Body mass index
BSA	Body surface area
CABG	Coronary artery bypass grafting
CI	Confidence interval
CKD	Chronic kidney disease
COPD	Chronic obstructive pulmonary disease
CRP	C-reactive protein
CT	Computed tomography
DM	Diabetes mellitus
EHR	Electronic health record
Hb	Hemoglobin
HTN	Hypertension
HF	Heart failure
HFrEF	Heart Failure with Reduced Ejection Fraction
HFmrEF	Heart Failure with Mid-range Ejection Fraction
HFpEF	Heart Failure with Preserved Ejection Fraction
ICH–GCP	International Council for Harmonisation–Good Clinical Practice
IRB	Institutional Review Board
KDIGO	Kidney Disease: Improving Global Outcomes
KAMC	King Abdullah Medical City
LOS	Length of stay
LVEF	Left ventricular ejection fraction
LVOT	Left ventricular outflow tract
MDCT	Multidetector computed tomography
MI	Myocardial infarction
MR	Mitral regurgitation
NYHA	New York Heart Association
OR	Odds ratio
PAD	Peripheral artery disease
PCI	Percutaneous coronary intervention
PPM	Permanent pacemaker
PVL	Paravalvular leak
RBBB	Right bundle branch block
RBC	Red blood cells
ROC	Receiver operating characteristic
SD	Standard deviation
SE	Self-expandable
SEV	Self-expanding valve
STS	Society of Thoracic Surgeons
TAVR	Transcatheter aortic valve replacement
TTE	Transthoracic echocardiography
VARC-3	Valve Academic Research Consortium-3
WBC	White blood cells
